# Prehospital Translation of Chest Pain Tools (RESCUE Study): Completion Rate and Inter-rater Reliability

**DOI:** 10.5811/westjem.2021.9.52325

**Published:** 2022-01-18

**Authors:** Anna C. Snavely, Simon A. Mahler, Nella W. Hendley, Nicklaus P. Ashburn, Brian Hehl, Jordan Vorrie, Matthew Wells, R. Darrel Nelson, Chadwick D. Miller, Jason P. Stopyra

**Affiliations:** * Wake Forest School of Medicine, Department of Emergency Medicine, Winston-Salem, North Carolina; † Wake Forest School of Medicine, Department of Biostatistics and Data Science, Winston-Salem, North Carolina; ‡ Wake Forest School of Medicine, Departments of Implementation Science and Epidemiology and Prevention, Winston-Salem, North Carolina; § Cape Fear Valley Health, Department of Emergency Medicine, Fayetteville, North Carolina

## Abstract

**Introduction:**

Chest pain is a common reason for ambulance transport. Acute coronary syndrome (ACS) and pulmonary embolism (PE) risk assessments, such as history, electrocardiogram, age, risk factors (HEAR); Emergency Department Assessment of Chest Pain Score (EDACS); Pulmonary Embolism Rule-out Criteria (PERC); and revised Geneva score, are well validated for emergency department (ED) use but have not been translated to the prehospital setting. The objectives of this study were to evaluate the 1) prehospital completion rate and 2) inter-rater reliability of chest pain risk assessments.

**Methods:**

We conducted a prospective observational cohort study in two emergency medical services (EMS) agencies (April 18, 2018 – January 2, 2019). Adults with acute, non-traumatic chest pain without ST-elevation myocardial infarction or unstable vital signs were accrued. Paramedics were trained to use the HEAR, EDACS, PERC, and revised Geneva score assessments. A subset of patients (a priori goal of N = 250) also had the four risk assessments completed by their treating clinicians in the ED, who were blinded to the EMS risk assessments. Outcomes were 1) risk assessments completion rate and 2) inter-rater reliability between EMS and ED assessments. An a priori goal for completion rate was set as >75%. We computed kappa with corresponding 95% confidence intervals (CI) for each risk assessment as a measure of inter-rater reliability. Acceptable agreement was defined a priori as kappa ≥ 0.60.

**Results:**

During the study period, 837 patients with acute chest pain were accrued. The median age was 54 years, interquartile range 43–66, with 53% female and 51% Black. Completion rates for each risk assessment were above goal: the HEAR score was completed on 95.1% (796/837), EDACS on 92.0% (770/837), PERC on 89.4% (748/837), and revised Geneva score on 90.7% (759/837) of patients. We assessed agreement in a subgroup of 260 patients. The HEAR score had a kappa of 0.51 (95% CI, 0.41–0.61); EDACS was 0.60 (95% CI, 0.49–0.72); PERC was 0.71 (95% CI, 0.61–0.81); and revised Geneva score was 0.51 (95% CI, 0.39–0.62).

**Conclusion:**

The completion rate of risk assessments for ACS and PE was high for prehospital field personnel. The PERC and EDACS both demonstrated acceptable agreement between paramedics and clinicians in the ED, although assessments with better agreement are likely needed.

## INTRODUCTION

Chest pain is the second most common reason patients come to the emergency department (ED) and accounts for 7–9 million patient visits to EDs in the United States every year.^1^,^2^ Many of these patients are transported by emergency medical services (EMS) and represent about 6–16% of prehospital patient encounters.^3^–^7^ Chest pain can signal life-threatening pathologies such as acute coronary syndrome (ACS) or pulmonary embolism (PE), or low-risk causes that do not need immediate intervention. Risk scores and care algorithms, such as the HEART pathway (history, electrocardiogram, age, risk factors, and troponin), are well-validated and commonly used in the ED to risk-stratify patients with chest pain.^8^–^11^ Although prehospital personnel have experience using algorithms as part of the evaluation of patients with trauma and strokes, chest pain risk-stratification tools have yet to be adopted in the prehospital setting.^12^–^15^

In a recent study, a modified prehospital HEART pathway used by paramedics demonstrated high negative and positive predictive value for adverse cardiac events. In that study, paramedics had access to point-of-care troponin measurement in the field and incorporated those results into their risk assessments.^16^ Currently, however, EMS use of point-of-care troponin in the US is limited to research studies.^16^,^17^ Thus, prehospital chest pain risk-stratification tools that do not require troponin measurement are needed. In addition, prehospital chest pain risk-stratification has been limited thus far to concern for acute coronary syndrome (ACS) and has ignored other life-threatening causes such as pulmonary embolism (PE).

Several candidate-risk assessments for both ACS and PE that do not require troponin results exist but have yet to be validated or compared in the prehospital setting. The HEAR (history, electrocardiogram, age, risk factors) score is an abridged version of the HEART pathway that is used to establish risk of major adverse cardiac events in patients with chest pain prior to or without troponin measurement. The ED Assessment of Chest Pain Score (EDACS) is another internationally validated tool for ACS that does not require troponin measurement.^9^ For the assessment of PE risk, the revised Geneva score risk-stratifies patients using risk factors, symptoms, and clinical signs to categorize patients as low, intermediate, and high probability.^11^ Pulmonary Embolism Rule-out Criteria (PERC) use eight variables to rule out PE in patients with a low pretest probability for PE.^10^

These chest pain risk-stratification tools have improved patient care in the ED, and their use could do the same in the prehospital setting by helping inform treatment and destination protocols. Furthermore, if very low-risk patients could be identified in the prehospital setting, these patients could avoid unnecessary transport to the ED. However, it is currently unclear which of these risk assessments, if any, are well suited for prehospital adoption. Therefore, the objectives of this study were to 1) evaluate the paramedic completion rate of each risk assessment and 2) evaluate the inter-rater reliability of the risk assessments between paramedics and clinicians in the ED.

**Population Health Research Capsule**

What do we already know about this issue?

*Chest pain is a common, high-stakes reason to call 911, but risk stratification tools have yet to be adopted in the prehospital setting.*

What was the research question?

*What is the completion rate and inter-rater reliability of chest pain risk assessments between paramedics and clinicians in the emergency department?*

What was the major finding of the study?

*While the completion rate of chest pain risk assessments was high, assessments with better agreement are likely needed.*

How does this improve population health?

*The incorporation of objective risk-stratification tools is feasible and may impact patient safety and system efficiency once incorporated into patient care protocols.*

## METHODS

### Design

This prospective, observational cohort study investigated four rapid risk-stratification tools to assess chest pain in the prehospital setting at two EMS agencies. The study was conducted over 8.5 months (April 18, 2018 – January 2, 2019). Participants were prospectively accrued under a waiver of informed consent. The study was approved by the institutional review board and registered with clinicaltrials.gov (NCT03494556). We used the Strengthening the Reporting of Observational studies in Epidemiology (STROBE) statement as a guide for reporting this observational study.

### Setting

The study was conducted by paramedics of two county EMS agencies in North Carolina with annual call volumes of approximately 75,000 and 14,000. These county-based agencies run paramedic/emergency medical technician crews and transport a majority of their patients to a single, tertiary medical center that provides regional coronary intervention capabilities. The agencies had prior experience with EMS process improvement projects and were partnered with a large, tertiary medical center. The individual EMS agencies shared a similar electronic health record (EHR) platform and quality assurance staff. Access to this system as well as to the medical system inpatient and outpatient EHR were readily available to EMS managerial staff for data collection.

### Population

Patients included in this study were at least 21 years old with acute, non-traumatic chest pain without ST-elevation myocardial infarction (STEMI) who were transported by ground EMS to a local ED. We excluded interfacility transports and patients with unstable vital signs (systolic blood pressure <90 millimeters mercury, heart rate >120 or <40, oxygen saturation <90% on room air or normal home oxygen flow rate).

### Risk Assessments

A total of 166 paramedics were trained to calculate the HEAR, EDACS, revised Geneva score, and PERC risk assessments. The HEAR and EDACS scores were chosen for their effectiveness and widespread use throughout US EDs. The PERC and revised Geneva score were selected because they are objective, simple, and do not require the paramedic to make diagnostic decisions, which would be outside their scope of practice. Paramedic training sessions included a two-hour, in-person orientation that reviewed ACS and PE pathophysiology, risk factors, and classic presentation characteristics. This was followed by inclusion and exclusion criteria and a description of the HEAR, EDACS, revised Geneva score, and PERC risk assessments and how they are used. The last part of the orientation was the application of assessments in multiple case-study simulations. The opportunity to review self-learning modules was made available to the paramedics throughout the study period. Training was completed one week prior to the start of recruitment. To complete risk assessments, paramedics used a standardized, one-page double-sided, data collection template with the risk assessments listed in the following order: HEAR score; EDACS score; revised Geneva score; and PERC risk assessment (Appendix 1). Prehospital risk assessments were not used to alter patient treatment or destination decisions. For a subset of patients (convenience sample), the emergency clinician also completed the four risk-stratification tools on a separate, but identical, standardized data collection form. Clinicians in the ED were blinded to EMS risk assessments. A sample size of 250 was chosen for the assessment of inter-rater reliability between EMS and ED assessments.

### Outcome Measures

The primary outcome was risk-assessment completion rate in all patients enrolled, and the secondary outcome was inter-rater reliability between EMS and ED assessments in the subset of patients where both assessments were collected. Based on prior experiences, EMS administrators set a goal to complete decision tools in >75% of eligible patients. Prehospital stroke scale assessments have been in use for many years and have completion rates of approximately 95%; thus, 75% was a reasonable estimate.^18^ However, while paramedics were encouraged to complete the decision aids, they were not informed of the 75% completion goal. Intermittent correspondence and in-person reminders were communicated to field personnel during the study period to encourage risk-assessment completion. To evaluate agreement between the paramedic and ED assessments, we calculated risk assessments based on the final scores recorded by the paramedic and the clinician in the ED for HEAR, EDACS and revised Geneva score. For PERC, the recorded risk assessment was used since there was no final score.

### Statistical Analyses

This study was designed to obtain precise estimates of completion rates (primary) and inter-rater reliability (secondary). With a total sample size of 800, the maximum half-width of an exact 95% confidence interval (CI) for completion rate was 0.035. In other words, each completion rate could be estimated +/− 3.5%. For a planned sample size of 250 with both EMS and ED assessments, and for an expected kappa ≥ 0.6, the maximum half-width of the 95% CI was 0.1 (ie, kappa could be estimated +/− 0.1). We estimated completion rates for each tool using the total number enrolled as the denominator and the number with a complete final score (HEAR, EDACS, revised Geneva) or risk assessment (PERC) as the numerator. Each tool’s completion rate was reported along with an exact 95% CI. We compared completion rates between the two ACS tools (HEAR and EDACS) and between the two PE tools (revised Geneva score and PERC) using chi-square tests.

To evaluate agreement between paramedic and emergency clinician, we categorized each assessment as low risk or non-low risk. For HEAR, a score of 0–3 was considered low risk, for EDACS, a score of less than 16 was considered low risk, and for revised Geneva, a score of 0–3 was considered low risk. For PERC, a patient was considered low risk if he or she met all the rule-out criteria. We evaluated agreement using both the kappa statistic and raw agreement. Acceptable agreement between the emergency clinician and paramedic was defined a priori as kappa ≥ 0.60. Both statistics were reported along with 95% CIs. We also used raw agreement to evaluate the agreement of individual components of each tool.

## RESULTS

**Figure 1:**
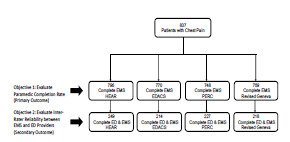
Study flow diagram. *ED,* emergency department; *EMS,* emergency medical services; *HEAR,* history, electrocardiogram, age, risk factors; *EDACS,* Emergency Department Assessment of Chest Pain Score;* PERC,* Pulmonary Embolism Rule-Out Criteria.

During the study period, a total of 837 patients with acute chest pain were accrued. The median age was 54 years, interquartile range (IQR) 43–66, with 53% female and 51% Black. A final score was completed on 95.1% of patients (796/837) for HEAR, on 92.0% (770/837) for EDACS, and on 90.7% (759/837) for revised Geneva score (Figure 1). For PERC a final risk assessment was completed on 89.4% (748/837) of patients. All completion rates exceeded the benchmark of 75%. Confidence intervals for each completion rate are provided in Table 1. When comparing the two ACS tools, a final score was completed more often for HEAR than EDACS (95.1% vs 92.0%, *P* = 0.01). Revised Geneva score and PERC had similar rates of completion for the assessment of PE (89.4% vs 90.7%, *P* = 0.4).

A total of 260 patients (31.1%; 260/837) were accrued who had assessments by both EMS and clinicians in the ED. In this subgroup the median age was 54 years (IQR 44–65), with 50% male and 52% Black, similar to the total enrolled population. Based on kappa, agreement between the paramedic and the emergency clinician was acceptable for EDACS (0.60; 95% CI, 0.49–0.72) and PERC (0.71; 95% CI, 0.61–0.81). However, agreement for HEAR (0.51; 95% CI, 0.41–0.61) and the revised Geneva score (0.51; 95% CI, 0.39–0.62) fell below the a priori definition of acceptability (0.6). Raw agreement was above 75% for all tools (Table 2). Cross-classification tables for EMS and ED assessments for each risk stratification tool are presented in Appendix 2. Raw agreement for each component of each ACS tool is presented in Table 3, and results for the PE tools are presented in Table 4.

## DISCUSSION

This study demonstrates that paramedics achieve high completion rates for chest pain risk-stratification tools, which suggests that implementation of these tools in the prehospital setting is highly feasible. Completion rates for HEAR, EDACS, revised Geneva score, and PERC risk assessments were all significantly higher than the 75% benchmark set a priori. The final score was completed more often for HEAR than EDACS, but this may have been the result of HEAR being the first risk-stratification tool on the data collection template. The HEAR score template used in this study is the longest score and built to improve objectivity, but this may have negatively impacted completion rate of the other scores. Completion rates of over 90% have been published within large ED cohorts.^8^ While completion rates over 75% are good, this rate would also likely increase once the risk score was included in protocol and directed clinical decision-making. In addition, the use of objective risk-stratification tools would further increase once standard treatment protocols change, quality improvement processes are initiated, and the paradigm of chest pain evaluation is shifted to rely on the use of risk-stratification tools.

In terms of agreement between EMS and clinicians in the ED, there was higher inter-rater reliability for EDACS and PERC (both meeting the bar of kappa ≥ 0.6), but agreement was less than acceptable for the HEAR and revised Geneva scores. When looking more closely at the ACS tools, agreement was fairly low for the history, ECG and risk factors components of the HEAR tool. The EDACS component that evaluates age in conjunction with either CAD or ≥ 3 risk factors showed low agreement as well. These components were all compound assessments, which likely negatively impacted agreement. Thus, it stands to reason that a tool that uses more single-answer assessments would provide improved agreement between healthcare personnel.

When developing or testing a risk-stratification tool, replicability is important. Training is frequently blamed when poor agreement is found, and this may be the case for ECG assessment within the HEAR score where agreement was only 53.1%. Interpretation of ECG is critical when evaluating patients for possible ACS. When paramedics completed the “E” part of the HEAR score, 64% gave a score of 0 (normal), whereas only 30% of clinicians in the ED gave a score of 0, suggesting that paramedics may be missing important ECG findings. This may be a result of a paramedic’s lack of experience differentiating acute ischemia from both non-specific and normal ECG tracings. Historically, paramedics receive rather focused training on the identification of STEMI, and there is very little emphasis on detecting more subtle ECG findings. The ability to use the HEAR score would be improved with additional training on ischemic and non-specific ECG findings. Alternatively, simplifying interpretations of ECGs into three categories – STEMI, abnormal but not a STEMI, and totally normal – could lead to improved inter-rater agreement.

For the two PE assessment tools, agreement was very strong for all of the components with the exception of heart rate. Agreement was higher for PERC where assessment of heart rate is yes/no (heart rate ≥ 100 at any time) than for the revised Geneva score where heart rate falls into three categories (<75, 75–94, > 95). This finding further supports the conclusion that single questions have better agreement when compared to compound or multiple category parts.

This study is an important first step in evaluating the prehospital use of objective, chest pain risk-stratification tools. The paradigm shift is dependent on completeness rates and agreement. They are important metrics to predict the ability to implement decision-aid use into clinical processes. Agreement between different categories of healthcare personnel has historically been poor.^19^,^20^ Our data is consistent with these findings. However, this should not limit the use of the tools but rather challenge us to adapt training or the tools themselves to improve agreement.

## LIMITATIONS

This study has several limitations. First, it is a prospective study that used a convenience sample for both the overall population enrolled and the subset with both ED and EMS assessments, which may have resulted in a selection bias. Information was not available on EMS calls where patients were not accrued to the study. The design included two EMS services that transported patients to a single medical center. Thus, the results of this study may not be generalizable to chest pain in other EMS systems or patients presenting to other institutions. It should be noted that our study design included two risk tools for ACS and two for PE. This was “double work” for our paramedics and likely negatively impacted completeness. The time to complete the assessments was not collected. A more pragmatic study would have selected one tool for ACS and one for PE and allowed them to inform clinical decisions.

Additionally, the order in which the tools were included in the data collection template completed by paramedics may have impacted completion rates. Agreement was compared between the paramedic and the emergency clinician, but no formal risk assessment training was provided for the clinicians. They likely were not experts in the use of these risk-assessment tools as there was no uniform tool or protocol to establish their use prior to this study. The assessments completed in the ED were assumed to be the most accurate as there was no gold standard exam for comparison, which impacts reliability and validity of paramedic assessments. Finally, the assessments performed by EMS and emergency clinicians were not done simultaneously. Patients frequently will provide different answers when questions are asked in different ways by different people at different times.

## CONCLUSION

High rates of completion of chest pain risk-stratification tools among paramedics in their usual patient care workflow suggest that decision-aid implementation is highly feasible. This is the first step in a paradigm shift in prehospital healthcare to empower objective decision-making in the care of patients with chest pain. The PERC and EDACS risk scores both had acceptable agreement between paramedics and clinicians in the ED based on the a priori definition of kappa ≥ 0.6. However, there is certainly room for improvement in agreement. Improvement may be achieved by modification of existing tools or a new prehospital tool that incorporates binary objective measures in the evaluation of both ACS and PE, as agreement was much better for simple objective criteria as compared to compound or subjective criteria. Further study is needed to evaluate the performance of these risk stratification tools and how they impact patient safety and system efficiency once incorporated into patient care protocols.

## Supplementary Information







## Figures and Tables

**Table 1:** Prehospital completion rates for each chest pain risk-stratification tool (N = 837).

Risk stratification tool	Paramedic final score % complete (95% CI)
HEAR	95.1% (93.4–96.5%)
EDACS	92.0% (89.9–93.7%)
PERC	89.4% (87.1–91.4%)
Revised Geneva Score	90.7% (88.5–92.6%)

*HEAR,* history, electrocardiogram, age, risk factors; *EDACS,* emergency department assessment of chest pain score; *PERC,* pulmonary embolism rule-out criteria; *CI,* confidence interval.

**Table 2:** Comparing emergency medical services (EMS) and emergency department assessments.

Risk stratification tool	Kappa (95% CI)	Raw agreement (95% CI)
HEAR (N = 249)	0.51 (0.41–0.61)	75.9% (70.1–81.1%)
EDACS (N = 214)	0.60 (0.49–0.72)	83.6% (78.0–88.4%)
Revised Geneva score (N = 218)	0.51 (0.39–0.62)	77.1% (70.9–82.5%)
PERC (N = 227)	0.71 (0.61–0.81)	87.7% (82.7–91.6%)

*HEAR,* history, electrocardiogram, age, risk factors; *EDACS,* Emergency Department Assessment of Chest Pain Score; *PERC,* Pulmonary Embolism Rule-Out Criteria; *CI,* confidence interval.

**Table 3:** Raw agreement for components of the acute coronary syndrome tools.

Component	Raw agreement (95% CI)
HEAR	
History	49.6% (43.2–56.0%)
ECG	53.1% (46.6–59.6%)
Age	90.3% (85.7–93.7%)
Risk factors	61.6% (55.2–67.8%)
EDACS	
Age	93.4% (89.2–96.4%)
Gender	92.6% (88.1–95.8%)
Age 18–50 and known CAD or 3+ risk factors	69.5% (62.8–75.7%)
Diaphoresis	82.4% (76.5–87.3%)
Pain radiates to arm or shoulder	75.7% (69.3–81.4%)
Pain occurred or worsened with inspiration	78.5% (72.3–83.8%)
Pain is reproduced by palpation	80.5% (74.5–85.6%)

*HEAR,* history, electrocardiogram, age, risk factors; *ECG,* electrocardiogram; *EDACS,* Emergency Department Assessment of Chest Pain Score; *CAD,* coronary artery disease; *CI,* confidence interval.

**Table 4:** Raw agreement for components of the pulmonary embolism tools.

Component	Raw agreement (95% CI)
PERC	
Age ≥ 50	95.6% (92.0–97.8%)
Heart rate ≥ 100 at any time	82.6% (77.0–87.3%)
Pulse oximetry on room air < 95% with good waveform	92.0% (87.6–95.2%)
Unilateral leg swelling	98.2% (95.5–99.5%)
Hemoptysis	98.7% (96.1–99.7%)
Recent surgery or trauma	99.1% (96.8–99.9%)
Prior PE or DVT	97.3% (94.2–99.0%)
Estrogen use	99.6% (97.5–100%)
Revised Geneva score	
Age > 65	94.5% (90.6–97.1%)
Previous PE or DVT	95.0% (91.2–97.5%)
Surgery under general anesthesia or lower limb fracture in the past month	97.7% (94.7–99.3%)
Cancer condition: current or considered cured within 1 year	97.3% (94.1–99.0%)
Unilateral lower limb pain	99.1% (96.7–99.9%)
Hemoptysis	98.2% (95.4–99.5%)
Heart rate	69.1% (62.5–75.2%)
Tenderness of lower limb deep-venous palpation AND unilateral edema	99.5% (97.4–100%)

*PERC,* Pulmonary Embolism Rule-out Criteria; *PE,* pulmonary embolism; *DVT,* deep venous thrombosis; *CI,* confidence interval.
