# Supplementary material for: Prehospital Translation of Chest Pain Tools (RESCUE Study): Completion Rate and Inter-rater Reliability
Source: West J Emerg Med. 2022 Jan 18;23(2):222–8. doi: 10.5811/westjem.2021.9.52325 (PMC8967468; doi:10.5811/westjem.2021.9.52325)
Supplement: Supplementary file 1 [file wjem-23-222-s001.docx]

**1. HEAR SCORE**

*See user guide for instructions*

**Patient Name____________________________________ DOB __________________ Event # ____________________**

| **History: (2 points max)** | | | |
| --- | --- | --- | --- |
| **High-Risk Features:**  Middle- or left-sided  Heavy/Tight/Pressure chest pain  Diaphoresis  Radiation  N/V  Exertional  Relief of symptoms by sublingual nitrates  **Low-Risk Features:**  Well localized  Sharp pain  Non-exertional  No diaphoresis  No N/V | Highly Suspicious  (Mostly high-risk features)  Moderately Suspicious  (Mixture of high-risk and low-risk features)  Slightly Suspicious  (Mostly low-risk features) | 2 Points  1 Point  0 Points | |
| **ECG: (2 points max)** | | | |
| ST-segment depression ≥ 1mm in two contiguous leads  T-wave inversion ≥ 1mm in two contiguous leads |  | 2 Points | |
| Non-specific changes  Repolarization abnormalities  Non-specific T wave changes  Non-specific ST-segment depression or elevation  Bundle branch blocks  Pacemaker rhythms  LVH (Left Ventricular Hypertrophy)  Early repolarization  Digoxin effect | Give 1 point total, not 1 point per abnormality | 1 Point | |
| Normal |  | 0 Points | |
| **Age: (2 points max)** | | | |
| ≥ 65  45-64  ≤44 |  | 2 Points  1 Point  0 Points | |
| **R**i**sk Factors: (2 points max)** | | | |
| **RISK FACTOR LIST:**  **Obesity (BMI ≥ 30)**  **Current or recent (≤ 90 days) smoker**  **Currently treated diabetes mellitus**  **Family history of CAD (1^st^ degree relative <55yo)**  **Diagnosed and/or treated hypertension**  **Hypercholesterolemia** | **Any** of the following:  🞏 Known Coronary Artery Disease (CAD)  🞏 Prior stroke  🞏 Peripheral Arterial Disease (PAD)  **OR**  🞏3 or more risk factors from list  1-2 Risk Factors from list | | 2 Points    1 Point |
| **Risk Level: *(select one)***  **🞏** **Low Risk (0-3 points)**  **🞏** **Not Low Risk (4+ points)** | No Risk Factors from list | | 0 Points |
| **HEAR Score (total points)**  Sum up points from all sections above  H+ E+ A+ R = total HEAR Score | | | ___________  Points |

| *See user guide for instructions*  🞏18-45 +2  🞏 46-50 +4  🞏 51-55 +6  🞏 56-60 +8  🞏 61-65 +10  🞏 66-70 +12  **E**  **D**  **A**  **C**  **S**  **Age** | ______ POINTS  ______ POINTS  ______ POINTS  ______ POINTS  ______ POINTS  ______ POINTS  ______ POINTS  🞏 71-75 +14  🞏 76-80 +16  🞏 81-85 +18  🞏 86+ +20 |
| --- | --- |
| Sex | 🞏 Male + 6 |
| 🞏 Known CAD  **OR**  Age 18-50 **AND** either:      🞏 ≥ 3 risk factors *(from list in HEAR Score)* | 🞏 Yes + 4 |
| Diaphoresis | 🞏 Yes + 3 |
| Pain radiates to arm or shoulder | 🞏 Yes + 5 |
| Pain occurred or worsened with inspiration | 🞏 Yes - 4 |
| Pain is reproduced by palpation | 🞏 Yes - 6 |

**__________**

**TOTAL POINTS**

**Risk Level: *(select one)***

**🞏 Low Risk** (< 16 points)

**🞏 Not Low Risk** (≥ 16 points)

| Age ≥ 50  **P**  **E**  **R**  **C** | 🞏 Yes |
| --- | --- |
| HR ≥ 100 at any time | 🞏 Yes  **Risk Level: *(select one)***  **🞏** **At Risk**  (any are “yes”)  **🞏** **Not at Risk**  (NONE are “yes”) |
| Pulse Ox on room air < 95% with good waveform | 🞏 Yes |
| Unilateral leg swelling | 🞏 Yes |
| Hemoptysis | 🞏 Yes |
| Recent surgery or trauma ≤ 4 weeks ago requiring general anesthesia | 🞏 Yes |
| Prior PE or DVT | 🞏 Yes |
| Estrogen use | 🞏 Yes |

______POINTS

______POINTS

_______ POINTS

______ POINTS

________ POINTS

_______ POINTS

____ POINTS

_______ POINTS

**R**ev

**G**

**E**

**N**

**E**

**V**

**A**

| Age > 65 | 🞏 Yes + 1 |
| --- | --- |
| Previous DVT or PE | 🞏 Yes + 3 |
| Surgery under general anesthesia **OR** lower limb fracture in past month | 🞏 Yes + 2 |
| Cancer condition- current or considered cured within 1 year | 🞏 Yes + 2 |
| Symptom of unilateral lower limb pain | 🞏 Yes + 3 |
| Hemoptysis | 🞏 Yes + 2 |
| Heart Rate | 🞏 ≥ 95 + 5  🞏 75-94 + 3  🞏 < 75 + 0 |
| Tenderness of lower limb deep venous palpation **AND** unilateral edema | 🞏 Yes + 4 |

**Risk Level: *(select one)***

**__________**

**TOTAL POINTS**

**🞏 Low Risk** (0-3 points)

**🞏 Intermediate Risk** (4-10 points)

**🞏 High Risk** (11+ points)

Paramedic Signature: ____________________________________________________ Date: ________________
