# Supplementary material for: Prehospital Translation of Chest Pain Tools (RESCUE Study): Completion Rate and Inter-rater Reliability
Source: West J Emerg Med. 2022 Jan 18;23(2):222–8. doi: 10.5811/westjem.2021.9.52325 (PMC8967468; doi:10.5811/westjem.2021.9.52325)
Supplement: Supplementary file 2 [file wjem-23-222-s002.docx]

Cross-Classification for EMS and ED Assessments for Each Risk Stratification Tool

| **HEAR (N=249)** | | |
| --- | --- | --- |
|  | **ED Non-Low Risk** | **ED Low Risk** |
| **EMS Non-Low Risk** | 117 | 14 |
| **EMS Low Risk** | 46 | 72 |
|  |  |  |
| **EDACS (N=214)** | | |
|  | **ED Non-Low Risk** | **ED Low Risk** |
| **EMS Non-Low Risk** | 44 | 11 |
| **EMS Low Risk** | 24 | 135 |
|  |  |  |
| **PERC (N=227)** | | |
|  | **ED Non-Low Risk** | **ED Low Risk** |
| **EMS Non-Low Risk** | 144 | 17 |
| **EMS Low Risk** | 11 | 55 |
|  |  |  |
| **Revised Geneva (N=218)** | | |
|  | **ED Non-Low Risk** | **ED Low Risk** |
| **EMS Non-Low Risk** | 54 | 34 |
| **EMS Low Risk** | 16 | 114 |

HEAR = History, Electrocardiogram, Age, Risk factors; EDACS = Emergency Department Assessment of Chest pain Score; PERC = Pulmonary Embolism Rule-out Criteria
